# Molecular determinants of acute kidney injury

**DOI:** 10.5249/jivr.v7i2.615

**Published:** 2015-07

**Authors:** Holger Husi, Christin Human

**Affiliations:** ^*a*^BHF Glasgow Cardiovascular Research Centre, University of Glasgow, Glasgow, UK.; ^*b*^Department of Hematology, Hemostasis, Oncology and Stem Cell Transplantation, Hannover Medical School (MHH), Hannover, Germany.

**Keywords:** Acute kidney injury, Glutamate, NMDA receptor, Death associated protein kinase, Systems biology

## Abstract

**Background:**

Acute kidney injury (AKI) is a condition that leads to a rapid deterioration of renal function associated with impairment to maintain electrolyte and acid balance, and, if left untreated, ultimately irreversible kidney damage and renal necrosis. There are a number of causes that can trigger AKI, ranging from underlying conditions as well as trauma and surgery. Specifically, the global rise in surgical procedures led to a substantial increase of AKI incidence rates, which in turn impacts on mortality rates, quality of life and economic costs to the healthcare system. However, no effective therapy for AKI exists. Current approaches, such as pharmacological intervention, help in alleviating symptoms in slowing down the progression, but do not prevent or reverse AKI-induced organ damage.

**Methods:**

An in-depth understanding of the molecular machinery involved in and modulated by AKI induction and progression is necessary to specifically pharmacologically target key molecules. A major hurdle to devise a successful strategy is the multifactorial and complex nature of the disorder itself, whereby the activation of a number of seemingly independent molecular pathways in the kidney leads to apoptotic and necrotic events.

**Results:**

The renin-angiotensin-aldosterone-system (RAAS) axis appears to be a common element, leading to downstream events such as triggers of immune responses via the NFB pathway. Other pathways intricately linked with AKI-induction and progression are the tumor necrosis factor alpha (TNF α) and transforming growth factor beta (TGF β) signaling cascades, as well as a number of other modulators. Surprisingly, it has been shown that the involvement of the glutamatergic axis, believed to be mainly a component of the neurological system, is also a major contributor.

**Conclusions:**

Here we address the current understanding of the molecular pathways evoked in AKI, their interplay, and the potential to pharmacologically intervene in the effective prevention and/or progression of AKI.

## Introduction

A number of tightly controlled and complex processes are performed by the heterogeneous cell populations of the kidney, from blood filtration by microvascular endothelial cells and podocytes, and reabsorption by proximal epithelial (tubular) cells to name a few. Acute kidney injury (AKI), also known as acute renal failure (ARF), is a common clinical event associated with a rapid loss of kidney function, leading to unacceptable high morbidity and mortality. ^1^ 0.4% to 0.6% of the total healthcare costs, between £400m and £600m, are annually spent on treatment for acute kidney injury in the UK alone and as many as 22% of hospitalized patients develop AKI.^2^ However, it is estimated that one-fifth of AKI that occurs after hospital admission is predictable and avoidable. ^3^

Over the last 10 years several international guideline groups have tried to establish consistent definitions and staging systems for AKI, namely the RIFLE (Risk, Injury, Failure, Loss, End stage kidney disease) system,^4^ which was modified by the AKIN (Acute Kidney Injury Network) group^5^ and further developed by KDIGO (Kidney Disease: Improving Global Outcomes). ^6^ The clinical assessment criteria for AKI are serum creatinine (SCr), blood urea nitrogen and urine output levels, whereby a rise in measurable quantities of SCr and blood urea associated with a decrease in urine levels is deemed indicative of AKI, especially if these occur rapidly within 48 hours.

However, it is apparent that a definitive and timely diagnosis using those measures has its limitations and hampers a reliable clinical assessment, which as a consequence can lead to serious diagnostic delays, potential misclassification of the actual injury status and stage, and offers a limited amount of information regarding the underlying cause. This in turn can result in a potential missed opportunity for therapeutic interventions at a point in time when kidney damage could be limitable or reversible. ^7^ Unmanaged or delayed action can lead to a number of complications, including metabolic acidosis, high potassium levels, uremia, changes in body fluid balance, and effects to other organs, and ultimately organ failure.^8^ This delay between assessment and clinical decision making has also been recognized as a potential reason for poor clinical outcomes often associated with AKI, ^9^ and it was suggested to use functional and injury biomarkers instead of, or in conjunction with, these clinical measures. ^10^

Nowadays, it has been widely recognized that AKI represents a multifactorial, heterogeneous syndrome, or a spectrum of diseases, that has the potential to be identified at an early stage, unlike the previous terminology of ARF which assumed that each AKI-induction pathway follows a similar or identical molecular route. ^11^ This also explains the sometimes contradictory research findings, but at the same time has the potential to confuse rather than solve the mystery surrounding AKI on a molecular level. Also, the identification of molecular events of renal impairment at an early stage would allow devising a suitable test that can be used for an immediate course of action to alleviate symptoms and disrupt the process of functional decline. ^12^ It is imperative that this condition is comprehensively understood on a molecular level to allow for targeted intervention therapies.

**Risk factors and triggers of AKI induction**

There is a considerable amount of clinical information available relating to observed AKI cases over the last 30 years that allows a wide-ranging analysis of AKI predisposition and causative agents. General risk factors are age greater than 65 years, heart failure, liver disease, diabetes, chronic kidney disease with or without diabetes, sepsis, urological obstruction, iodinated contrast agents, nephrotoxic medication and hypovolaemia/shock.^13^ AKI can be induced by many different events such as rapid blood loss to and from the kidney, vasoconstrictive drugs, exposure to harmful substances, hypotension linked to sepsis, and obstruction of the urinary tract. Table 1 lists the main factors that can lead to AKI,^14^ where surgical procedures or medication are often precursors.

**Table 1:** Events leading to AKI induction.

Site	Trigger
Pre-renal	- Volume depletion due to hemorrhage, severe vomiting or diarrhea, burns
	- Edema due to cardiac failure, cirrhosis, nephrotic syndrome
	- Hypotension due to cardiogenic shock, sepsis, anaphylaxis
	- Cardiovascular due to severe cardiac failure, arrhythmias
	- Renal hypoperfusion induced by non-steroidal anti-inflammatory drugs (NSAIDs) or specific enzyme inhibitors or receptor blockers involved in the renin-angiotensin axis, abdominal aortic aneurysm, renal artery stenosis or occlusion, hepatorenal syndrome
Renal	- Glomerular disease due to inflammation (glomerulonephritis), thrombosis, hemolytic uraemic syndrome
	- Tubular injury due to acute tubular necrosis following prolonged ischaemia, and nephrotoxins such as aminoglycosides, radiocontrast media, cisplatin, heavy metals
	- Acute interstitial nephritis due to drugs (e.g. NSAIDs), infection or autoimmune diseases
	- Vascular disease including vasculitis, cryoglobulinaemia, polyarteritis nodosa, thrombotic microangiopathy, cholesterol emboli, renal artery stenosis, renal vein thrombosis, malignant hypertension
	- Eclampsia
Post-renal	- Urinary tract obstructions due to Calculus formation (i.e. kidney stones), urethral stricture, prostatic hypertrophy or malignancy, blood clot
	- Papillary necrosis
	- Bladder tumor
	- Radiation and retroperitoneal fibrosis
	- Pelvic malignancy

Such a vast array of events leading to the same outcome makes it impractical to devise general strategies to combat the onset of AKI using clinical measures alone. Nevertheless, this can be substantially improved if common and reliable indicators of tissue damage at an early stage can be demonstrated, especially if non-invasive tools, such as the use of molecular biomarkers, can be employed.

**Biomarkers of AKI**

Currently, the main clinical objective regarding AKI is the prevention of disease onset due to the lack of suitable and effective treatment and the irreversibility of organ damage. Monitoring disease onset and progression usually involves clinical markers such as SCr levels. However, SCr levels are known to be influenced by factors other than renal causes alone, lack the power of prediction due to its nature of being modulated after kidney irregularities and show low sensitivity. ^15^ Other traditional clinical biomarkers of kidney injury observed in blood (urea nitrogen) and urine (epithelial cells, tubular casts, fractional excretion of Na+, urinary concentrating ability, etc.) have also been demonstrated to be insensitive and nonspecific for the diagnosis of AKI, ^16^ and therefore such measurements are a poor marker of acute deterioration in kidney function.^17^ This led to the search of potentially more reliant biomarkers, and over the last decade a considerable amount of studies were performed to identify specific molecular biomarkers which could replace or augment the value of the current physiological markers.

Promising diagnostic injury markers include kidney injury molecule 1 (KIM-1), ^16^ interleukin 18 (IL-18), ^18^ neutrophil gelatinase-associated lipocalin (NGAL), ^19^ liver-type fatty acid binding protein (L-FABP), ^20^ cystatin C, ^21^ N-acetyl-beta-D-glucosaminidase (NAG), ^22^ beta-2-microglobulin (B2M) ^23^ zinc-alpha-2-glycoprotein (AZGP1), ^24^ and cytochrome C. ^25^ Furthermore, several additional biomarkers have been postulated to be of prognostic or diagnostic value in connection with AKI (reviewed in e.g.)^10,17,26,27^

KIM-1 is a promising marker for various renal diseases as well as AKI, which includes but not limits to tubular necrosis, since AKI incidence precedes any rise of the conventional SCr.^28^ KIM-1 expression is undetectable in normal kidneys, whereas the mRNA and protein levels are markedly up-regulated in AKI. ^29^

Urinary IL-18 was reported to be significantly elevated in patients one to two days prior to an observed rise in SCr and confirmed AKI diagnosis. ^18^ It has been shown to perform better as a predictive biomarker in children, ^30^ and multiple preclinical studies demonstrated that IL-18 is not only a biomarker but also a mediator of ischemic AKI. ^31^

NGAL is expressed during systemic inflammation and sepsis,^15^ and was found to be highly increased post-intervention in patients undergoing cardiac surgery and subsequently developing AKI compared to non-AKI patients undergoing the same treatment. ^32^ It was shown in a cohort of more than 500 intensive care patients that it has a moderate prediction value for a predisposition to develop AKI. ^33^ NGAL as a clinical marker is now in clinical trial phase and undergoing prospective evaluation after it was shown to be in accordance with altered serum creatinine levels as well as biopsy results in adult AKI patients. ^34^

L-FABP has been reported to be elevated in non-diabetic CKD^16^ and in established AKI of diverse causes, including acute tubular necrosis, sepsis, and nephrotoxin exposure. ^35^

Cystatin C, a marker for glomerular filtration rates that is freely reabsorbed by the glomerulus and catabolized by the tubulus, and was shown to be elevated in tubular dysfunction.^16^ It was proposed to be an early onset marker for AKI in urine ^36^ and serum.^37^

Urinary NAG is a molecule shed from the proximal tubules into the urine. It is a proximal tubular damage marker and correlates with the grade of injury, and indeed has been shown to correlate with various diseases and toxic agents affecting the kidneys, but also other diseases including rheumatoid arthritis and hyperthyroidism creating doubts on its specificity.^16^

B2M, the light chain of the major histocompatibility complex (MHC) I, which is present on every living cell, is shed normally but also usually reabsorbed by the proximal tubular cells. In tubular damage situations it becomes present in urine, which has been shown in surgery and transplantation studies.^16,27^

Serum levels of AZGP1, best known as a marker in cancer progression, were demonstrated in a small study to be increased in the early phase of AKI, and high initial levels of AZGP1 correlated with extra-renal complications but not with parameters of renal function.^24^

Elevated levels of extracellular cytochrome C is a bona fide indicator of cell death burden in any organ or tissue, and is released during mitochondrial damage as an initiator of apoptosis, necrotic cell lysis and oxidative stress. It was observed in drug-induced AKI,^25^ and holds potential as a marker for necrotic conditions.

A recent meta-analysis of medium- to large-scale clinical/prospective studies indicated that the biomarkers serum cystatin C and urinary IL-18 and NGAL showed the best performance for early diagnosis of AKI, serum cystatin C, urine IL-18 and KIM-1 were indicative for the differential diagnosis of established AKI, and levels of urine NAG, KIM-1, and IL-18 performed the best for mortality risk prediction after established AKI.^17^ Some of these biomarker have already been proven to be elevated in patients developing AKI in different clinical settings such as patients in intensive care units (ICU), before and after heart or other surgery, in diabetic or obese patients. Currently, however, they are not able to replace the conventional measured values like GFR, cystatin C, creatinine and urea. There is also hope that these novel biomarkers will discriminate between the underlying pathophysiology of AKI (i.e. toxins, sepsis, ischemia or multifactorial), and will enable to distinguish AKI from other renal disease such as chronic kidney disease.^9^

A different approach to not only determine key factors and molecular modulators, but also pinpoint druggable targets, is the elucidation of molecular events such as downstream signaling cascades involved in renal insults.

**Molecular hallmarks and modulated pathways**

AKI can have many seemingly unrelated initiators, however commonalities converge on the affected organ, thereby enabling a much better view of how kidney damage is inflicted on a molecular level. It is suggested that renal cell loss, secondary to metabolic, genetic, immune, toxic, oxidant, and other mechanisms, is a common determining factor that can result in a broad spectrum of clinical renal syndromes.^38^ Molecular hallmarks of AKI are hyperglycaemia, vasoconstriction, accumulation of free and esterified cholesterol, activation of the renin-angiotensin-aldosterone system (RAAS), inflammation and inflammatory response, altered tubule dynamics leading to increased luminal sodium, hypoxia, cellular ATP depletion, renal apoptosis and necrosis (Table 2).^39^

**Table 2:** Hallmarks of AKI.

Hallmark *	Modulated associated event
RAAS activation	↑ Angiotensin signaling
Na+/Cl- retention, increased luminal Na+	↑ Aldosterone/cortisol signaling events
Hyperglycemia	↑ Diabetes
Tubular cell dynamics	↑ Infiltration of immature cells
Cytoskeletal reorganization	↑ ECM remodeling
Elevated blood pressure	↑ Hypertension
Accumulation of free and esterified cholesterol	↑ Systemic stress response
PI3K modulation	↓ Phosphoinositol-3-kinase activity
	↓ Insulin signaling
Vasoconstriction	↑ Vasoconstrictors (endothelin, angiotensin, MMP2)
	↓ Vasodilators (nitric oxide NO)
Hypoxia	↑ Hypoxia inducing factor HIF1α
	↑ NADPH oxidases
	↑ ROS levels
	↑ NFkB activity
	↑ Inflammation factors (TNFα, TF, PAT1, MCP1)
	↑ Inflammation and inflammatory response
	↑ Atherogenesis, fibrinogenesis
	↓ ATP levels
	↓ NAD levels
	↑ Hypoxanthine levels
	↑ Necrosis

* Clinical and disease model observations are listed based on modulated associated events, and the arrows represent either up- or down-regulation.

The major signaling cascades involved in AKI are the RAAS axis, tumor necrosis factor alpha (TNF α ), transforming growth factor beta (TGF β ), epidermal growth factor receptor (EGFR), hypoxia inducing factor (HIF1α) and NFkB pathways.^40,41^ A recent study integrated a majority of the various elements of AKI, delineated all potentially involved pathways and assembled them into a global molecular model of AKI induction, progression and ultimately apoptosis/necrosis using a combination of Systems Biology, proteomics and de-novo pathway mapping approaches. ^42^ Additionally, a dataset from a proteomic study of chemically induced AKI was established in this investigation and used to remove unsupported pathways, thereby uncovering the involvement of the glutamatergic system in renal damage.

**Figure 1:**
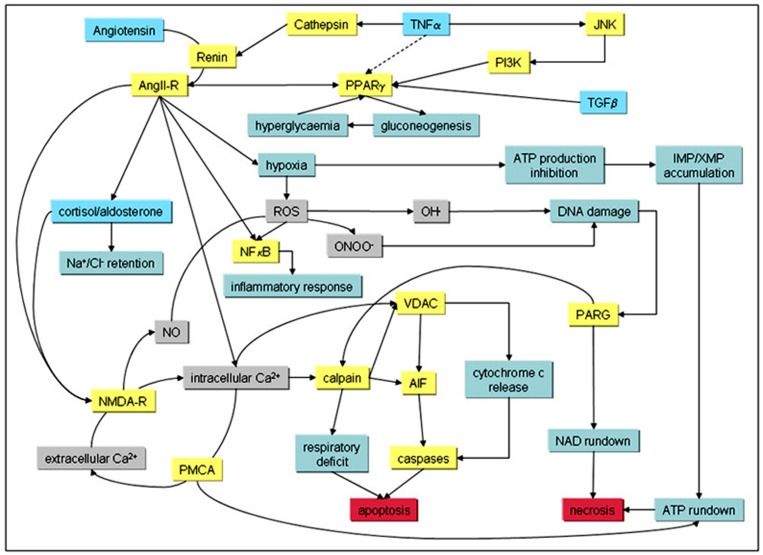
Signaling cascades and evoked pathways involved in AKI. Known primary modulators of AKI (blue boxes) with downstream targets (yellow boxes) and hallmarks (green boxes) are shown. Grey boxes denote chemical compounds, and red boxes indicate endpoints in AKI.

A potential summary of AKI-modulated pathways and signaling events leading to Ca^2+^-overload and apoptosis as well as necrosis in kidney tissue after injury is shown in Figure 1.

Potentially initiating factors such as TNFα and other cytokines lead to activation of the RAAS cascade,^43,44^ however induction of AKI can occur via multiple stimulation or entry points, ^45^ including vasoconstriction, which also activates the angiotensin receptor. ^46^ These events lead to the activation of peroxisome proliferator-activated receptor gamma (PPARγ ), ^47^ which is involved in the expression/inhibition of gluconeogenesis proteins. ^48^ This can then lead to hyperglycemia via glycogen phosphorylase activity. ^49^ Hyperglycemia has also been shown to be chemically induced by 20-hydroxyeicosatetraenoic acid (20-HETE) which involves the cAMP/protein kinase A-phosphorylase kinase-glycogen phosphorylase pathway.^50^

Vasoconstriction, one of the hallmarks of AKI, is induced by RAAS,^51^ and has been shown to lead to hypoxic conditions, ^52^ which in turn triggers the asymmetric gene activation of elements involved in the glycolysis pathway such as phosphofructokinase (PFKL).^53^ An accumulation of the PFKL metabolic product fructose 1,6-bisphosphate can result in the inhibition of de novo ATP production and ultimately accumulation of hypoxanthine, ^54^ which is another known hallmark of AKI.

Other downstream events of RAAS and hypoxia are the activation of the NFkB signaling cascade^55^ leading to inflammatory responses,^56^ and the cortisol/aldosterone receptor pathway, resulting in Na+/Cl- -retention.^57^ The potential role of the functionally similar NFkB2 has been postulated, ^41^ but remains currently unclear.

The activation of the RAAS axis also leads to a raise in intracellular Ca^2+^, either by targeting intracellular Ca^2+^-stores or induction of the glutamatergic system involving the N-methyl-D-aspartate (NMDA) receptor (NMDA-R). The latter might also require the production of reactive oxygen species (ROS) in a NADPH oxidase (NOX)-dependent pathway^58^ under hypoxic conditions,^59^ leading to gene activation of the NMDA-R.^60^ It could also be shown that death associated protein kinase 1 (DAPK1), which is a gene activation target of the JNK-p53 system, is up-regulated in AKI.^42^ This gene expression can also occur via TGFβ -mediated SMAD induction,^61^ and has been shown to inhibit NFkB activation by TNFα -induced apoptosis.^62^ Additionally, the NMDA-R is a known target of DAPK1, where DAPK1-mediated channel modulation results in a permanently open NMDA-R, potentially leading to an uncontrollable elevation of intracellular Ca^2+^, nitric oxide (NO) and ROS, and ultimately to non-reversible cell death. ^63^ This is directly associated with nuclear DNA damage, mitochondrial apoptotic pathway activation via calpain and caspase activation, and poly(ADP-ribose) glycohydrolase (PARG)-mediated necrosis involving molecular events described above.^64,65^

Additionally, molecules involved in cytoskeletal reorganization, cell integrity and extracellular matrix have also been shown to be induced in AKI, and their up-regulation was suggested to be a cellular protective mechanism from angiotensin II- and high-glucose-induced apoptosis.^66^

**The renal glutamatergic system**

**Figure 2:**
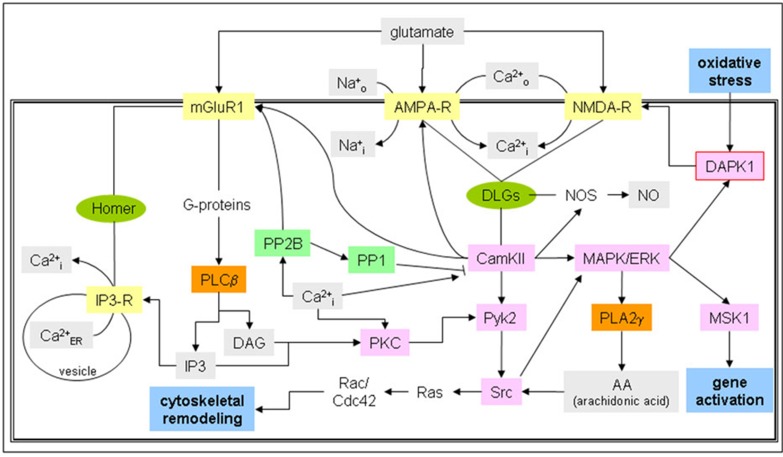
Glutamatergic signaling cascades in renal tissue. Glutamate receptors NMDA-, AMPA- and metabotropic mGluR1 receptors, which are present and functional in tubular cells as well as podocytes, are depicted with potential associated signaling cascades ranging from calcium signaling to phospholipase and adenylate cyclase cascades and their interlinking pathways. Metabolites are depicted with a grey box, receptors in yellow, kinases in pink, phospholipases in orange, and phosphatases with a green box. Global parameters are shown with a blue box and scaffolders with a green ellipse.

Scant information is available on the involvement of the glutamatergic system in kidney function. The majority of molecular studies performed involving the glutamatergic system are in the area of neuroscience, where it could be demonstrated that different types of glutamate receptors are coupled to specific molecular signaling cascades.^67^ The NMDA-R in particular was shown to be tightly integrated in a vast molecular network, ^68^and surprisingly many of these components were also not only found in kidney tissues, but also in conjunction with the NMDA-R up-regulated after AKI induction. ^42^ The relevant endogenous NMDA-R ligand in AKI has not been characterized, however kidney glutamate levels are increased in AKI,^69^ and expression of these proteins in tubular cells has been shown.^70^ A recent study has shown that glutamate and NMDA-R polyamine binding site agonist spermidine aggravated oxidative stress and ischemia-reperfusion-induced AKI,^71^ reasoning that glutamate itself is the renal activator of the glutamatergic system. A simplified diagram of this system is shown in Figure 2.

Renal receptors for glutamate modulated in AKI are the metabotropic glutamate receptor 1 (mGLUR1), α- amino-3-hydroxy-5-methyl-4-isoxazolepropionic acid (AMPA) receptor (AMPA-R) and the NMDA-R. ^42^ mGluR1-signaling occurs via G-proteins, resulting in the activation of phospholipase C beta (PLCB). ^72^ Thus, the intracellular Ca^+2^-release channel IP3-R, which is linked to this system via the mGluR1-scaffolder Homer, ^73^ can be directly activated. ^74^ This release of vesicular Ca^2+^ can activate calcineurin (PP2B), which is able to block calcium/calmodulin-dependent protein kinase type II (CamKII) indirectly via protein phosphatases 1 (PP1). ^75,76^ CamKII is a downstream target of NMDA-R activation,^77^ and is coupled to this receptor via scaffolders belonging to the disks large family DLGs, ^78^ that also bind to the Ca^2^+/Na+-importer channel AMPA-R. ^79^ Nitric oxide synthase (NOS) is also bound to DLGs ^80^ and activated by CamKII, ^81^ thereby linking NO-production directly to NMDA-R activity. CamKII is also involved in protein tyrosine kinase 2 (Pyk2) ^82^ and mitogen activated protein kinase/extracellular signal-regulated kinase (MAPK/ERK)^83^ cascade activation, and both of those events can lead to a substantial number of diverse signaling end-points through various other cascades and molecules involved. One example of converging signaling is tyrosine-protein kinase Src that can be activated by Pyk2 phosphorylation, ^84^ arachidonic acid (AA) modulation via phospholipase A 2 gamma (PLA2γ )^85^ downstream of MAPK/ERK, ^86^ or diacylglycerol (DAG) induction by PLC β and PKC activity, ^87^ and can lead to the activation of the Ras/Rac/Cdc42 pathway. ^88,89^ MAPK/ERK signaling can also result in gene activation events through phosphorylation of MSK1, ^90^ and directly activates DAPK1,^91^ which in turn targets the NMDA-R. The NMDA-R has also been shown to be involved in cytoskeletal reorganization in neurons,^92^ but whether this is also the case in renal cells is currently unknown. However, the required molecular components are present in these cells.

It has to be noted that this glutamatergic system might not exist in its entirety in a single cell, but is dispersed across several cell types, whereby ionotropic glutamate receptors and its associated signaling components might be expressed in one type of cells, and the metabotropic receptor and associated signaling pathways in another.

**Prevention and pharmacological intervention**

A considerable number of pharmacological or other intervention studies were performed to date due to the clinical importance of AKI. Summarily, inhibition of molecules linked to the TNFα -dependent modulation in the AKI-induced pathways, including ROS production, hyperglycemia and specific NFkB-dependent signaling cascades, all had an improving or attenuated outcome (see overview of pharmacological intervention studies in).^42^

Inhibition of TNFα signaling by blocking the cognate receptor using anti-TNFα antibodies resulted in prevention of the induction of AKI.^93^ Targeting the penultimate step of Ca^2+^-overload by inhibiting NMDA-R using the channel blocker D-AP5 was shown to significantly reduce ischemia/reperfusion injury (I/RI) -induced glomerular and tubular dysfunction,^94^ and raises the possibility that NMDA receptor signaling is one of the penultimate steps prior to non-reversible apoptosis and necrosis. However, inhibition of the NMDA-R in the clinical setting could be problematic due to the intricate involvement of this receptor in physiological processes occurring in neuromuscular junctions (e.g. heart) as well as cognitive functions.^95^ Nonetheless the NMDA-R channel blocker Mg^2+^ has been successfully used in the clinic management of pre-eclampsia and eclampsia. ^96^

Another molecule which gained considerable attention was sphingosine kinase-1 (SK1). It catalyzes the phosphorylation of sphingosine to form sphingosine 1-phosphate (S1P), which in turn stimulates and promotes activation of NFkB in response to TNF signaling and thereby diverting the AKI-induced stimulation of deleterious inflammation.^97^ It could be demonstrated that interleukin 11 (IL-11), which is an approved chemotherapy-induced thrombocytopenia treatment, induces this molecule via a HIF1-α dependent pathway, resulting in powerful renal protective effects by reducing necrosis, inflammation, and apoptosis in ischemia-induced AKI.^98^ Isoflurane administration in I/RI-induced AKI could also be shown to have the same downstream target as IL-11, where SK1 is induced through ERK MAPK activation, and resulted in protection against renal endothelial apoptosis. ^99^

Targeting the receptor of S1P has also shown great promise in AKI treatment. Using S1P as a pre-treatment prior to AKI induction in an I/RI animal model resulted in an attenuation of systemic inflammation and kidney injury,^100,101^ and the S1P receptor type 2 (S1P(2)R) antagonist JTE013 selectively up-regulated SK1 and attenuated both hydrogen peroxide-induced necrosis and TNFα/cycloheximide-induced apoptosis.^102^

The compound TDZD-8, which targets and inhibits glycogen synthase kinase-3β, protects against endotoxemic acute renal failure mainly by down-regulating pro-inflammatory TNF-α and RANTES. It was shown to ameliorate NSAID-induced AKI by induction of renal cortical COX-2 and direct inhibition of the mito-chondrial permeability transition. ^103,104^

An as yet untested likely druggable target in AKI-associated symptoms could be DAPK1 due to its direct link with the NMDA-R. This molecule has been shown to be a prospective target in cancer treatment by exploiting its potential apoptotic action, however inhibition studies by chemical compounds are somewhat lacking. Nevertheless, one recent report of designed kinase inhibitors using octahedral metal chelate complexes with a ruthenium(II) or iridium(III) metal center demonstrated a highly selective octasporine protein kinase inhibitor termed OS4 with a DAPK1-IC50 of 2nM.^105^ Such a compound deserves further investigation and shows a promising way forward in combating AKI-induced tissue injury.

The results of pharmacological intervention studies reported in the literature suggest that AKI mediated tissue damage effects can be reduced and in principle even prevented or to some degree reversed, and a combination of various drugs, targeting specific AKI-induced pathways and molecules, might potentially be a line of attack in disease prevention or therapeutic intervention.

## Conclusion

AKI currently remains a serious issue in clinical care and intervention therapies, however substantial advances in understanding the symptoms on a molecular level will undoubtedly lead to an improved way to devise novel therapy regimes and/or detection methods, and currently the best route of action is to prevent the occurrence of AKI in the first place, close monitoring of renal function and alleviating symptoms if kidney damage has occurred.

Current research in AKI biomarker discovery has gone from strength to strength over the preceding years and it is only a matter of time to find the right marker(s) for each of the various causes of AKI. While potential proteinaceous molecular markers are under heavy investigation and close scrutiny, one should not ignore chemical metabolic markers of AKI, where prospective studies have not gained the same level of attention. These types of biomarkers might hold great potential to rapidly and reliably assess kidney damage in the clinical setting.

Therapeutic regimes by pharmacological intervention in AKI prevention or progression have already yielded outstanding results and will quite likely be the best course of action. However, many compounds tested to treat or prevent AKI symptoms are unknown to be effective in humans, still need to be tested in larger cohorts, or approved to be used in the clinical setting. Future research for novel compounds could also be driven by exploiting the molecular processes involved in AKI. This requires a better understanding of molecular events in the various facets leading to AKI symptoms.

The current trend in AKI disease management, coupled with a global effort to not only alleviate symptoms, but effectively combat AKI and challenge its root causes, holds great promise to tackle this issue head on and succeed in the not too distant future.
